# Psychometric evaluation of the Arabic version of the 5-item Problem Areas in Diabetes (AR-PAID-5) scale

**DOI:** 10.1186/s12875-022-01758-z

**Published:** 2022-06-09

**Authors:** Hazem A. Sayed Ahmed, Samar F. Mohamed, Mona Mostafa, Sally Fawzy Elotla, Asghar Shah, Jaffer Shah, Ahmed Mahmoud Fouad

**Affiliations:** 1 Department of Family Medicine, Faculty of Medicine, Suez Canal University, Ismailia, Egypt; 2 Department of Internal Medicine, Faculty of Medicine, Suez Canal University, Ismailia, Egypt; 3 Department of Public Health, Occupational and Environmental Medicine, Faculty of Medicine, Suez Canal University, Ismailia, Egypt; 4 Division of Biology and Medicine, Brown University, Providence, RI USA; 5 Medical Research Center, Kateb University, Kabul, Afghanistan

**Keywords:** Diabetes distress, PAID-5, Primary healthcare, Type 2 diabetes mellitus

## Abstract

**Background:**

Screening for diabetes distress is recommended when caring for patients with type 2 diabetes mellitus (T2DM) in primary healthcare (PHC). The 5-item Problem Areas in Diabetes (PAID-5) scale is widely used to measure diabetes distress, but its Arabic validation studies are scarce, so this study was carried to assess the psychometric properties of the Arabic version of the PAID-5 (AR-PAID-5) in Egyptian PHC patients with T2DM.

**Methods:**

We conducted a cross-sectional study including 260 participants from six rural PHC settings in Ismailia governorate, Egypt. Internal consistency using Cronbach’s α and one-month test-retest reliability using intraclass correlation coefficient (ICC) were investigated. Confirmatory factor analysis (CFA) was used to evaluate the one-factor structure of the AR-PAID-5. Correlations of the AR-PAID-5 with the Arabic versions of the 20-item Problem Areas in Diabetes (PAID), Patient Health Questionnaire 9 (PHQ-9), Generalized Anxiety Disorder 7 (GAD-7), 5-item World Health Organization Well-Being Index (WHO-5) scales and glycated hemoglobin (HbA1c) were investigated for supporting the convergent validity. Associations of the PAID-5 with sociodemographic, and clinical characteristics were assessed for demonstrating the discriminant validity. Criterion validity was also evaluated.

**Results:**

There was a good internal consistency (α = 0.88) and a stable test-retest reliability (ICC = 0.74). The CFA confirmed the one-factor structure of the AR-PAID-5. Significant positive correlations existed between the AR-PAID-5 with diabetes distress evaluated by the Arabic version of the PAID (rho = 0.93, *p* < 0.001), depressive symptoms (PHQ-9) (rho = 0.56, *p* < 0.001), anxiety symptoms (GAD-7) (rho = 0.47, *p* < 0.001), emotional well-being (WHO-5) (rho = − 0.38, *p* < 0.001), and HbA1c (rho = 0.16, *p* = 0.003). A satisfactory discriminant validity, and an acceptable criterion validity were demonstrated.

**Conclusions:**

The AR-PAID-5 scale is a reliable and valid tool that can be used for diabetes distress screening and in research in Arabic speaking PHC patients with T2DM.

## Introduction

The International Diabetes Federation estimates the global diabetes prevalence to be 463 million people (9.3%) in 2019 [1]. There is a projected 96% increase in diabetes incidence in the Middle East and North Africa, from 55 million in 2019 to an estimated 108 million in 2045. Type 2 diabetes mellitus (T2DM) accounts for 90% of diabetes globally [2].

Physical activity, proper dosing of medication, and blood glucose level monitoring are important factors in the self-management of diabetes, and may pose a negative or otherwise burdensome emotional experience on some patients [3, 4]. This emotional burden that is associated with diabetes is termed diabetes distress [5]. Diabetes distress is one of the most prevalent (36%) and important psychosocial barriers to efficacious care for people with diabetes [6, 7]. Conclusions from a 6-month prospective study indicated that improving diabetes distress may improve diabetes quality of life in young women with diabetes [8].

The American Diabetes Association recommends assessment for symptoms of diabetes distress among diabetic patients using appropriate standardized and validated tools at their initial visit, at periodic intervals, and when there is a change in disease, treatment, or life circumstance [9]. Several psychometric scales have been developed to assist clinicians in ascertaining a patient’s diabetes distress. The most frequently used, diabetes-specific, and clinical-evidence based scales in determining diabetes distress are the 20-item PAID scale and 17-item Diabetes Distress Scale (DDS17) [5, 6, 10, 11]. PAID is a self-report survey consisting of a 20-item questionnaire rated from 1 being “Not a problem” to 4 being “Serious problem” [5]. This original PAID scale has demonstrated reliability and validity in the English-speaking population [12].

McGuire and colleagues developed psychometrically robust short versions of the PAID, PAID-5 and 1-item PAID (PAID-1). The PAID-5 is composed of five of the emotional-distress questions from the PAID-20 items (items 3, 6, 12, 16, and 19) while the PAID-1 is composed of item 12 from the PAID. The PAID-5 is reliable and valid tool, is useful in rapid screening, poses a lesser burden on patients [13], and is a widely used tool [6] despite the development of the 2-item DDS and the 4-item DDS [14].

As the burden of diabetes and utility of determining diabetes distress exists globally and across language, efforts have been made to translate and validate the PAID and associated shortened versions. The PAID and PAID-5 were translated into Korean (K-PAID and K-PAID-5). The short form K-PAID-5 performed better on psychometric evaluations (known-groups validity, internal-consistency, and test–retest reliability) than the K-PAID, and the authors conclude that the short form may be beneficial in imposing a minimum hardship on patients with diabetes [15].

The Norwegian translated version of the PAID-5 was demonstrated to be reliable and valid in assessing diabetes distress among people with both type 1 and type 2 diabetes [16]. The Turkish version of the PAID-5 demonstrated satisfactory convergent validity, but its reliability and discriminative validity were not reported (17). A telephone survey was conducted to evaluate the psychometric properties of the German multi-item instruments. Authors demonstrated a good internal reliability of the German PAID-5, and at least mediocre fit for a one-factor model, however convergent and discriminative validity were not evaluated [17].

Prior to this study, there was no Arabic short form of the PAID scale. Given the importance of ascertaining diabetes distress, and the predicted rise in diabetes in the Middle East, a translated, validated AR-PAID-5 is in order. As such, this study is the first to evaluate the psychometric properties of the AR-PAID-5 in PHC patients with T2DM in Egypt.

## Methods

### Design, sampling and setting

This cross-sectional study was conducted on a sample of patients with T2DM attending the PHC settings in Ismailia governorate, Egypt, between September 2020 and June 2021. A convenience sampling strategy was used to collect data from eligible patients in the 6 rural PHC settings affiliated with the Egypt’s Ministry of Health and Population. We used the Soper’s online calculator of sample size for structural equation models, to estimate the required sample size for a CFA model of one-latent and five observed variables, given that the PAID-5 has five items [18, 19]. A calculated sample size of 234 was large enough to detect an anticipated effect size of 0.061 [16] at 5% alpha error and 80% power of the study (additional 10% of the calculated sample size was added to compensate for dropout). Accordingly, the required sample size was 260 patients.

Patients aged 18 years or older were eligible to participate in this study if they had been diagnosed with T2DM for at least 1 year, and patients provided their informed consent to participate. Patients with gestational diabetes and those who were not able to give their informed consent due to serious mental illness or cognitive impairment were excluded. Data collection was performed using face-to-face interviews by the co-first author. To examine test-retest reliability of the AR-PAID-5 scale, data of the retest questionnaire were collected from 100 participants one-month after the first assessment.

### Study measures and scales

The initial part of the study questionnaire included questions about sociodemographic and clinical characteristics including: age, gender, marital status, occupation, family income, duration of diabetes, treatment for diabetes, diabetes-related long-term complications (e.g. cardiovascular, cerebrovascular, retinopathy, nephropathy, neuropathy, or peripheral vascular complications), and smoking. Further parts of the study questionnaire included the Arabic versions of the following scales: PAID [5], PAID-5 [13], PHQ-9 [20, 21], GAD-7 [21, 22], and WHO-5 [23, 24].

The original PAID scale was developed in English and consisted of 20 items scored on a 5-point Likert scale, ranging from 0 to 4; where 0 = not a problem, and 4 = serious problem. The PAID gives a total score range of 0 to 100, by summing the 20 items’ responses and multiplying this sum by 1.25. The higher scores indicate greater diabetes-related emotional distress, with a score of ≥40 indicating severe emotional distress [25, 26]. The PAID-5 includes questions 3, 6, 12, 16 and 19 of the original PAID scale. PAID-5 gives a total score ranging from 0 to 20, with a score of ≥8 indicating high diabetes-related distress [13].

The Arabic version of the PAID (AR-PAID) was obtained from Joslin Diabetes center. We conducted a validation study of the Joslin’s AR-PAID on 200 patients with T2DM. Cronbach alpha was 0.96 and test-retest reliability demonstrated stability (ICC = 0.97) [27]. CFA demonstrated fit to the four-factor model of the Spanish PAID [28]. Convergent and discriminant validity were satisfactory displayed.

PHQ-9 is the depression module of the full PHQ. It consists of 9 items; each item is scored from 0 (not at all) to 3 (nearly every day), with a total score ranging from 0 to 27. A cut-off value ≥10 had a sensitivity of 88% and a specificity of 88% for major depression [20]. The Arabic version of the PHQ-9 (AR-PHQ-9) is available and showed satisfactory validity and reliability [21]. GAD-7 is the anxiety module of the full GAD consisting of 7 items. Each GAD’s item can be scored from 0 (not at all) to 3 (nearly every day), with a total score ranging from 0 to 21. A cut-off point ≥10 indicating GAD (sensitivity: 89%, specificity: 82%) [22]. The Arabic version of the GAD-7 (AR-GAD-7) is available with satisfactory validity and reliability [21].

WHO-5 is among the most widely used questionnaires assessing subjective psychological well-being [29]. It was originally presented at a WHO meeting in Stockholm in February 1998 as part of a project on the measurement of well-being in PHC patients [30] and was derived from the WHO-10 [23]. This scale only contains positively phrased items. The degree to which these feelings were present in the last 2 weeks is scored on a 6-point Likert-type scale ranging from 0 (not present) to 5 (constantly present). Item scores are summated and transformed to a 0–100 scale, multiplying the raw score by 4 [31]. A valid and reliable Arabic version of the WHO-5 (AR-WHO-5) was developed in an elderly population in Lebanon [24].

Anthropometric measurements including body weight (kg) and height (cm) were measured in all participants. Body mass index (BMI) was calculated as the body weight (in kg) divided by height in meters squared. Participants with BMI values ≥30 kg/m^2^ were categorized as obese, while BMI values of 25–29.9 were considered overweight. The most recent HbA1c values (less than 8 weeks prior or 12 weeks after interviewing the patient) were used. HbA1c values less than 7% and 7.5% were used to identify adult and older adult patients with good glycemic control, respectively [9].

### Ethical consideration

The study procedures were approved by the Research Ethics Committee on Human Studies in accordance with the Declaration of Helsinki, at the Faculty of Medicine, Suez Canal University, Ismailia, Egypt under reference number 4277/2020. Informed consent was obtained from all participants. All methods were carried out in accordance with the Research Ethics Committee’s guidelines and regulations.

### Statistical analysis

The Statistical Package for the Social Sciences (SPSS), version 25.0 (IBM Corporation, NY, USA) was used to perform all data management and analyses, while Mplus software, version 7.4 was used to conduct the confirmatory factor analysis (CFA) [32]. A significance level of 0.05 was used in all statistical analyses. All categorical variables were summarized as frequencies and percentages (%). The distributions of continuous variables were tested for normality with the Kolmogorov Smirnov test. Median and interquartile range were used for non-normally distributed variables.

The reliability of the AR-PAID-5 scale was assessed by internal consistency (Cronbach’s α) and the test-retest reliability (ICC). A CFA with robust weighted least squares estimator used to investigate the factor structure of the AR-PAID-5. Model fit was assessed by goodness-of-fit measures: ratio of Chi-square [χ^2^] value to the degrees of freedom [df] (CMIN/DF) and associated *p* values, goodness-of-fit index (GFI), comparative fit index (CFI), Tucker Lewis Index (TLI), root mean squared error of approximation (RMSEA) and standardized root mean square residual (SRMR). The model fit was considered acceptable if the following criteria were satisfied: CMIN/DF < 3, CFI ≥0.90, TLI ≥0.90, and RMSEA ≤0.08 [15, 33].

Convergent validity was assessed by Spearman’s Rank-Order Correlation (rho) between diabetes-related emotional distress (AR-PAID-5) and depressive symptoms (AR-PHQ-9), anxiety symptoms (AR-GAD-7) and the level of glycemic control (HbA1c). Correlation between the AR-PAID-5 and PAID scales was also investigated. According to Cohen’s conventions to interpret effect size, a correlation coefficient of < 0.30 is small/ weak, 0.30–0.49 moderate, and 0.50 or more is large/strong [34].

Discriminant validity was used to determine whether the AR-PAID-5 scale can differentiate between groups of patients with depression/anxiety symptoms, poor glycemic control as well as other demographic and clinical variables. Independent-samples Mann-Whitney and Kruskal Wallis test (Since these data were not normally distributed) were used to assess discriminant validity. Criterion validity was evaluated using receiver operating characteristic (ROC) curves with high diabetes-related distress as the external criterion met by a cut-off value of ≥33 on the PAID [13, 25]. Youden index-based optimal cut-off value for the AR-PAID-5 was identified along with its area under curve (AUC), sensitivity, specificity, positive predictive value (PPV) and negative predictive value (NPP).

## Results

### Descriptive statistics

This study included a sample of 260 patients with T2DM. The mean age of the patients was 48.3 years (±11.4 years) (range 25–80 years), and females constituted 56.5% of the sample. The majority were either married, divorced or widowed. About one-fourth of the sample were either illiterate or did not complete secondary education degree, while about half of the sample were either not employed or retired. The mean duration of T2DM was 7.8 years (±5.5 years) (range 1–30 years), with about one-third of less than 5-year duration. Two-thirds of patients were on oral hypoglycemic agents while 34.2% were on an insulin-containing regimen. Seventy patients (26.9%) had a single diabetes-related complication while 34.6% had two or more complications. The most frequent diabetes-related complications in our sample were: neuropathy (50.8%), retinopathy (38.1%), foot problems (30.4%), and nephropathy (24.2%). Other chronic comorbidities included obesity (32.7%), hypertension (20.8%), and dyslipidemia (8.1%). The mean HbA1c was 7.8% (±0.68%) (range 6–10.5%), with only 26 patients (10%) achieved the glycemic control target (Table 1).

**Table 1:** Patients’ demographic and clinical characteristics (*N* = 260)

Characteristics	Frequency (%)
**Age** (years), mean ± SD (range)	48.3 ± 11.4 (25–80)
Less than 40 years	65 (25.0%)
40–59	142 (54.6%)
60+	53 (20.4%)
**Gender**	
Female	147 (56.5%)
Male	113 (43.5%)
**Marital status**	
Single	10 (3.8%)
Married	197 (75.8%)
Divorced/ widow	53 (20.4%)
**Education level**	
Illiterate	55 (21.2%)
Less than secondary	8 (3.1%)
Secondary	158 (60.8%)
University and above	39 (15.0%)
**Work status**	
Not Employed/ Housewives	111 (42.7%)
Employed/Business owners/Freelancers	120 (46.1%)
Retired	29 (11.2%)
**Duration of diabetes**, mean ± SD (range)	7.8 ± 5.5 (1–30)
Less than 5 years	85 (32.7%)
5–10 years	111 (42.7%)
More than 10 years	64 (24.6%)
**Type of antidiabetic medications**	
Oral hypoglycemics	171 (65.8%)
Insulin-containing regimens	89 (34.2%)
**Number of diabetes-related complications**	
None	100 (38.5%)
Single	70 (26.9%)
Two or more	90 (34.6%)
**Type of diabetes-related complications**	
Retinopathy	99 (38.1%)
Nephropathy	63 (24.2%)
Cardiovascular	3 (1.2%)
Neuropathy	132 (50.8%)
Foot problems	79 (30.4%)
Others	18 (6.9%)
**Other chronic comorbidities**	
Obesity	85 (32.7%)
Hypertension	54 (20.8%)
Dyslipidemia	21 (8.1%)
**HbA1c %**, mean ± SD (range)	7.8% ± 0.68% (6.0–10.5%)
**Glycemic control**	
Controlled	26 (10.0%)
Uncontrolled	234 (90.0%)

*SD* Standard deviation, *HbA1c* Glycated hemoglobin

#### Reliability of the AR-PAID-5: internal consistency and test-retest reliability

The means and standard deviations of the AR-PAID-5 items are described in Table 2. Inter-item correlations for the AR-PAID-5 scale ranged from 0.47 to 0.69, while item-to-total correlations ranged from 0.70 to 0.85. Cronbach’s α for the AR-PAID-5 scale was 0.88. The test-retest reliability of the AR-PAID-5 was measured in 100 patients who gave their repeated questionnaires, with an ICC of 0.74 (95% CI: 0.61–0.83, *p* < 0.001).

**Table 2:** Internal consistency and test-retest reliabilities of the AR-PAID-5

Items of the AR-PAID-5	Mean (SD) (*N* = 260)	Inter-items correlations (*N* = 260)					Test-retest reliability (*N* = 100)	
		item 3	item 6	item 12	item 16	item 19	ICC	95% CI
**Item 3**	0.71 (1.29)	1.00	–	–	–	–	0.98	0.97–0.99
**Item 6**	0.67 (1.21)	0.69 ***	1.00	–	–	–	0.98	0.97–0.99
**Item 12**	1.85 (1.48)	0.49 ***	0.47 ***	1.00	–	–	0.96	0.94–0.97
**Item 16**	1.08 (1.26)	0.56 ***	0.58 ***	0.54 ***	1.00	–	0.94	0.91–0.96
**Item 19**	1.18 (1.40)	0.56 ***	0.52 ***	0.54 ***	0.61 ***	1.00	0.74	0.63–0.82
**Total AR-PAID-5**	**5.49 (5.50)**	0.71 ***	0.70 ***	0.85 ***	0.78 ***	0.81 ***	**0.74**	**0.61–0.83**
		**Cronbach α = 0.88**						

Item 3 = “Feeling scared”; item 6 = “Feeling depressed”; item 12 = “Worrying about future complications”; item 16 = “Feeling that diabetes is taking up too much energy”; item 19 = “Coping with complications”

*AR-PAID-5* Arabic version of the 5-item Problem Areas in Diabetes, *SD* Standard deviation, *ICC* Intra-class correlation, *CI* Confidence interval

***. Spearman’s Rank-Order Correlation is statistically significant at *p*-value < 0.001

### Validity of the AR-PAID-5

#### Construct validity: factor structure of the AR-PAID-5

**Fig. 1:**
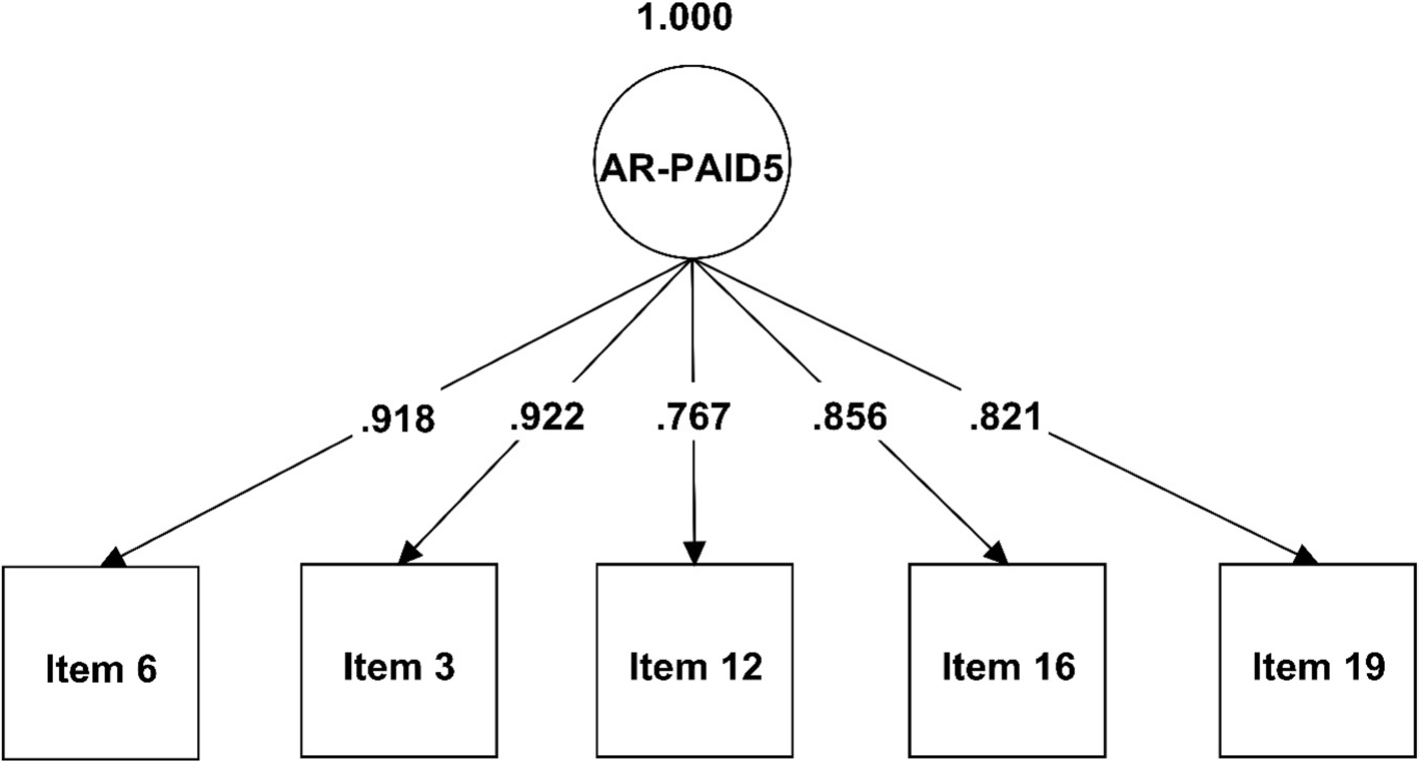
A Path diagram illustrating the factor structure of the AR-PAID-5 with item-specific factor loadings

A CFA was performed for the AR-PAID-5 and illustrated in Fig. 1 and Table 3. A hypothesized single-factor model was used for AR-PAID-5. The overall model fit, Chi-square was significant (χ^2^ = 11.5, df = 5, CMIN/DF = 2.3, *p*-value = 0.042), denoting that the model was not exactly fit. However, other model fit indices were excellent (CFI =0.987, TLI = 0.995, SRMR = 0.022 and RMSEA = 0.071). Factor loadings representing the hypothesized item-to-scale relationships were also satisfactory and statistically significant, and ranged from 0.767 to 0.992.

**Table 3:** Factor loadings of the AR-PAID-5 from the confirmatory factor analysis (*N* = 260)

Items of the AR-PAID-5	Factor loadings		
	Standardized estimate	SE	*p*-value
**Item 3**	0.922	0.020	0.000*
**Item 6**	0.918	0.020	0.000*
**Item 12**	0.767	0.034	0.000*
**Item 16**	0.856	0.021	0.000*
**Item 19**	0.821	0.030	0.000*
	**Goodness-of-fit indices**		
**Model fit χ**^**2**^ (df, *p*-value)	11.516 (5), 0.042		
**CMIN/DF**	2.3		
**CFI**	0.997		
**TLI**	0.995		
**SRMR**	0.022		
**RMSEA**	0.071		

Item 3: “Feeling scared”; item 6: “Feeling depressed”; item 12:Worrying about future complications”; item 16: “Feeling that diabetes is taking up too much energy”; item 19: “Coping with complications”

CMIN/DF, ratio of Chi-square [χ^2^] value to the degrees of freedom [df] (good if CMIN/DF < 3); CFI, comparative fit index (good fit ≥0.90); TLI, Tucker Lewis Index (good if ≥0.90); SRMR, standardized root mean square residual (good fit ≤0.08); RMSEA, root mean square error of approximation (acceptable fit ≤0.08); SE, standard error

*. Statistically significant at *p*-value < 0.05

#### Convergent validity of the AR-PAID-5

There was a significant positive and strong correlation between the total scores of AR-PAID-5 and the AR-PAID (rho = 0.94, *p* < 0.001). Significant correlations also existed between all AR-PAID-5 items and the total AR-PAID; ranging from 0.72 to 0.84. Convergent validity of the AR-PAID-5 was also confirmed by a significant strong correlation with the AR-PHQ-9 scale for depression (rho = 0.56, *p* < 0.001), and a significant moderate correlation with the AR-GAD-7 scale for anxiety (rho = 0.47, *p* < 0.001). The AR-PAID-5 total score also demonstrated a significant moderate inverse correlation with the AR-WHO-5 (rho = − 0.38, *p* < 0.001). Higher scores on the AR-PAID-5 were associated with higher scores on the AR-PHQ-9 and AR-GAD-7 scales. Higher scores on the AR-PAID-5 were associated with lower scores on the AR-WHO-5. However, the AR-PAID-5 total score showed a weak significant correlation with HbA1c (rho = 0.16, *p* = 0.003), Table 4.

**Table 4:** Correlations between the AR-PAID-5 and the Arabic versions of the PAID, PHQ-9, GAD-7, WHO-5 and HbA1c (*N* = 260)

Items of the AR-PAID-5	Correlation coefficient (*p-*value)				
	AR-PAID	AR-PHQ-9	AR-GAD-7	AR-WHO-5	HbA1c
**Item 3**	0.65 ***	0.44 ***	0.34 ***	−0.34 ***	0.20 **
**Item 6**	0.69 ***	0.41 ***	0.35 ***	−0.31 ***	0.13 *
**Item 12**	0.82 ***	0.47 ***	0.47 ***	−0.37 ***	0.16 *
**Item 16**	0.71 ***	0.44 ***	0.31 ***	−0.36 ***	0.09
**Item 19**	0.71 ***	0.46 ***	0.36 ***	−0.20 ***	0.09
**Total AR-PAID-5**	**0.93** ***	**0.56** ***	**0.47** ***	−0.38 ***	**0.16** **

*AR-PAID-5* Arabic version of the 5-item Problem Areas in Diabetes, *AR-PAID* Arabic version of the Problem Areas in Diabetes, *AR-GAD-7* Arabic version of the Generalized Anxiety Disorder Scale 7, *HbA1c* Glycated hemoglobin, *AR-PHQ-9* Arabic version of the Patient Health Questionnaire 9, *AR-WHO-5* Arabic version of the 5-item World Health Organization Well-Being Index

***. Spearman’s Rank-Order Correlation is statistically significant at *p*-value < 0.001; **. Statistically significant at *p*-value < 0.01; *. Statistically significant at *p*-value < 0.05

#### Discriminant validity of the AR-PAID-5

AR-PAID-5 scale discriminated well between diabetes-related emotional distress levels among patients with different demographic and clinical characteristics (Table 5). Female sex, older age, and longer disease duration were significantly associated with high scores on AR-PAID-5. Patients on an insulin-containing regimen had higher AR-PAID-5 scores, compared to patients on oral hypoglycemic agents.

**Table 5:** Associations between the AR-PAID-5 and patients’ sociodemographic and clinical characteristics (*N* = 260)

Characteristics	n	The AR-PAID-5 score		***p***-value
		Mean (±SD)	Median (IQR)	
**Age** (years)				
Less than 40	72	4.60 (±4.9)	4.0 (0–7.0)	0.000*
40–59	150	4.65 (±4.7) ^a^	4.0 (1.0–7.0)	
60+	38	8.83 (±6.9) ^a^	6.0 (4.0–15.0)	
**Gender**				
Female	149	6.33 (±5.6)	5.0 (2.0–9.0)	0.001*
Male	111	4.41 (±5.2)	3.0 (0–6.0)	
**Duration of diabetes** (years)				
Less than 5	97	4.20 (±4.4)	3.0 (0–7.0)	0.000*
5–10 years	107	4.76 (±4.9)	4.0 (1.0–6.0)	
More than 10	56	8.48 (±6.6) ^a, b^	6.0 (3.5–14.0)	
**Type of antidiabetic medications**				
Oral hypoglycemics	171	4.49 (±4.5)	4.0 (1.0–7.0)	0.001*
Insulin-containing regimens	89	7.42 (±6.6)	5.0 (2.0–11.0)	
**Number of diabetes-related complications**				
None	100	3.38 (±4.1)	2.0 (0–5.0)	0.000*
Single	70	5.08 (±4.7) ^a^	4.0 (2.0–8.0)	
Two or more	90	7.56 (±6.2) ^a^	6.0 (3.0–10.0)	
**Other chronic comorbidities**				
Obesity	85	6.33 (±5.5)	5.0 (3.0–8.0)	0.019*
Hypertension	54	8.85 (±7.2)	6.0 (3.0–17.0)	0.000*
Dyslipidemia	21	10.43 (±7.6)	11.0 (4.0–18.0)	0.003*
**Glycemic control **				
Good	23	5.27 (±5.4)	4.0 (1.0–8.0)	0.768
Poor	237	5.52 (±4.7)	4.5 (0–9.0)	
**Depressive symptoms (PHQ-9 ≥ 10)**				
No	250	4.75 (±4.7)	4 (1.0–7.0)	0.000*
Yes	10	14.40 (±6.5)	18 (6.5–20.0)	
**Anxiety symptoms (GAD-7 ≥ 10)**				
No	241	5.19 (±5.2)	4 (1.0–8.0)	0.000*
Yes	19	13.89 (±7.0)	19 (7.0–20.0)	
**Well-being (WHO-5 ≤ 50**)				
Good	215	5.13 (±5.20)	4 (1.0–8.0)	0.043*
Poor	45	7.24 (±6.55)	5 (2.0–10.0)	

*AR-PAID-5* Arabic version of the 5-item Problem Areas in Diabetes, *GAD-7* Generalized Anxiety Disorder Scale 7, *PHQ-9* Patient Health Questionnaire 9, *WHO-5* 5-item World Health Organization Well-Being Index, *IQR* Interquartile range

*Statistically significant at *p*-value < 0.05

^a^Statistically significant difference compared to the first category; b. Statistically significant difference compared to the second category

The number of diabetes-related complications was significantly and positively associated with AR-PAID-5 scores, where patients with single or multiple complications had higher scores than those with no complications. Furthermore, patients who had obesity, hypertension or dyslipidemia had significantly higher AR-PAID-5 scores compared to patients without these comorbidities. Nevertheless, there was no significant difference in AR-PAID-5 scores between patients with glycemic control and those uncontrolled (*p* = 0.768). Known-group validity was confirmed by the statistically significant differences in AR-PAID-5 score between patients with symptoms of depression/anxiety (i.e. PHQ-9/GAD-7 scores of ≥10); and poor wellbeing (i.e. WHO-5 wellbeing index ≤50).

#### Criterion validity: estimation of the AR-PAID-5 cut-off value for high diabetes-related emotional distress

**Fig. 2:**
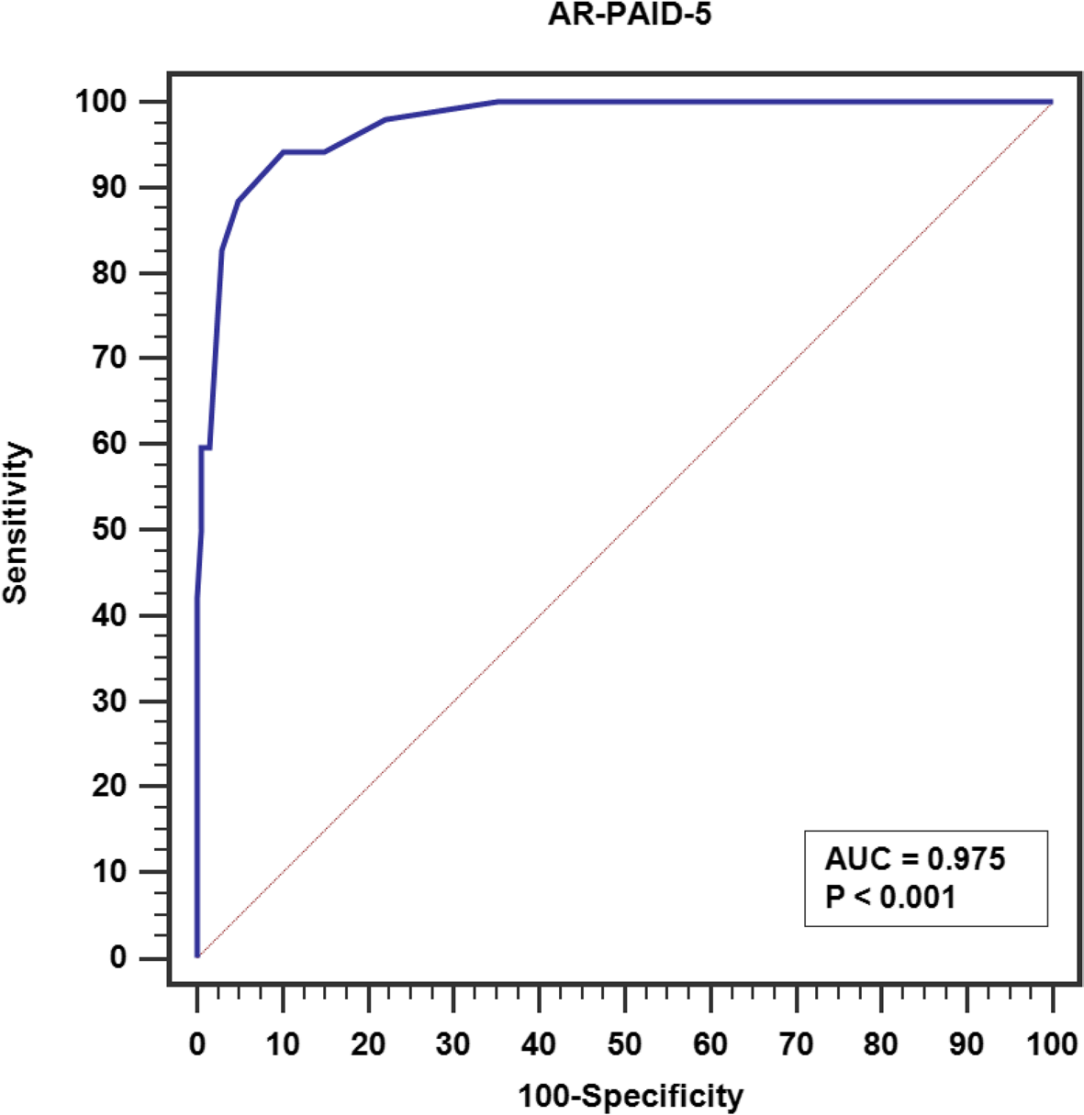
Receiver Operating Characteristic (ROC) curve of the AR-PAID-5 for high diabetes-related emotional distress (*N* = 260)

Criterion validity of the AR-PAID-5 was assessed using ROC curve with high diabetes-related emotional distress as an external criterion met by a cut-off value ≥33 on PAID-20. AR-PAID-5 had an excellent diagnostic accuracy for high diabetes-related emotional distress, confirmed by an AUC value of 0.975 (95% CI: 0.95–0.99, *p* < 0.001), Fig. 2. A Youden index-based optimal cut-off value for the AR-PAID-5 score was ≥8 with a sensitivity of 88.4% (95% CI: 76.6–95.6), a specificity of 95.2% (95% CI: 91.3–97.7), a PPV of 82.1% (95% CI: 71.4–89.5) and a NPV of 97.1% (95% CI: 94.0–98.6).

## Discussion

This was the first study to assess the psychometric properties of the AR-PAID-5 in primary care patients in the Middle East and North Africa region. The results of the present study show satisfactory psychometric properties of the AR-PAID-5 in PHC patients with T2DM. Our findings demonstrated good reliability, a satisfactory construct validity, a confirmed convergent validity, a well-discriminant validity, and a good criterion validity of the AR-PAID-5.

The AR-PAID-5 possesses good internal consistency, and a stable test-retest reliability. Our findings are in line with the internal consistency’s results of the original PAID-5, K-PAID-5, Norwegian PAID-5, and German PAID-5 studies, which demonstrated Cronbach’s α varying from 0.83 to 0.89. The test-retest reliability of our study is acceptable despite the fact that the ICC is less than the findings in previous studies (0.81–0.89) [15, 16], the test-retest reliability of the original PAID-5 and the German PAID-5 scales were not evaluated [13 18].

Our findings showed an excellent construct validity of the AR-PAID-5, the CFA verified the one factor structure of this scale with excellent goodness-of-fit measures, which is in congruence with measures seen in previous studies [13, 15, 16]. Our used CFA model did not need modification with covariance of errors while both of the Korean and Norwegian studies demonstrated excellent goodness-of-fit measures after model modification with the covariance of error terms between two items (item 3, and 6 items; 3 and 16, respectively) [15, 16]. In the Germany study, CFA showed mixed results of model fit, which did not fully confirm the one-factor structure of the original PAID-5 [13, 17].

In our study, the AR-PAID-5 total score correlated positively with symptoms of diabetes distress (AR-PAID) depressive symptoms (AR-PHQ-9), anxiety symptoms (AR-GAD-7), and HbA1c level, and negatively with emotional well-being, indicating a reasonable convergent validity. In the present study, correlation between the AR-PAID-5 score and the AR-PAID score was significantly positive and strong, this finding is similar to the reported findings in the original PAID-5 and the Norwegian version of the PAID-5 [13, 16].

Our study revealed a moderate positive correlation of the AR-PAID-5 total score with depressive symptoms. This finding supported the findings from the two previous validation studies of the PAID-5 [15, 16]. This finding is partially similar to the reported findings in other studies on the PAID, which showed moderate to strong positive correlations with different measures of depression [15, 35–37].

Our study also demonstrated a moderate positive correlation between the AR-PAID-5 total score and anxiety symptoms. This finding is congruent with findings of previous studies on the PAID scale [27, 38]. Snoek et al. reported that a bidirectional relationship between anxiety and diabetes distress seems to be present, but this issue has received very little attention. Anxiety symptoms are characterized by excessive worries and fear about several situations. Thus, it cannot be difficulty seen a phenomenological overlap with the concept of diabetes distress as captured with the PAID-5, with phrases like feeling scared, and worrying [39]. This is the first study to assess this relationship during validation process of the PAID-5 scale.

The present study found a moderate negative correlation between the AR-PAID-5 and the WHO-5 well-being index. McGuire et al. and Vislapuu et al. found weak negative correlations between these two questionnaires [13, 16]. Unsurprisingly, when diabetes stress symptoms increase well-being of the diabetic patients decreases [13, 16, 31].

Our results showed a weak positive correlation between the AR-PAID-5 total score and a higher HbA1c level. This finding is consistent with the previous studies on the PAID-5 [15, 16], and the PAID scales [12, 15, 17, 36, 39–42]. Furthermore, a previous study revealed that diabetes distress had a strong positive correlation with HbA1c.level among patients with T2DM, in which diabetes distress was evaluated by the DDS 17 scale [43]. Diabetes distress may have an adverse effect on HbA1c level through its contribution to impaired diabetes self-care behaviours, the presence of comorbid depression, and dysregulation of stress hormones [39].

The current study showed that the AR-PAID-5 scale is able to distinguish between patients’ diabetes distress with most of the demographic and clinical characteristics. Known-groups validity also revealed differences in the AR-PAID-5 score between patients with more or less depression/anxiety symptoms, and emotional well-being indicating discriminant validity. Hermanns et al. concluded that the PAID may be useful screening tool for diabetes distress and depression [25].

The present study showed that patients with poor glycemic control had higher diabetes distress scores than patients with good glycemic control, but association between diabetes distress and achieving glycemic control targets was non-significant. This may be related to the relative few numbers of patients, who achieved glycemic control targets.

The present study revealed that the AR-PAID-5 scale discriminated between groups, such as patients with and without, obesity, hypertension or dyslipidemia. The AR-PAID-5 scores were significantly associated with older age, longer disease duration, and number of diabetes-related complications.

The AR-PAID-5 total score was significantly associated with receiving insulin regimen. The Norwegian version of the PAID-5 have not significantly found associations with insulin alone, insulin with oral hypoglycemic agents or oral hypoglycemic agents alone. The relatively small sample size might be the reason of that study’s finding, in addition to the possibility of treatment type was not the main reason for participants’ diabetes distress [16]. Use of insulin therapy was significantly associated with the PAID score in previous studies [42, 44–46]. The negative emotional response of patients towards receiving insulin therapy through the course of diabetes is referred as insulin distress, which is not only a part of diabetes distress, but it is unique identity [47].

Female patients had significantly higher diabetes distress scores than male patients. McGuire et al. and Lee et al. found a similar finding [13, 15]. Vislapuu et al. found that female patients reported higher scores on the Norwegian version of the PAID-5 than male patients, but without a significant difference [16]. The vulnerability of females to diabetes distress might be related to socio-demographic, biological and cultural factors. The rising demands of diabetes self-care might be more challenging for women than man as they often have multiple child-rearing and household support roles and responsibilities in traditional societies [41]. Depression and anxiety among patients with T2DM also are associated with female gender and the existing of these psychological problems also is associated with increased symptoms of diabetes distress [39, 48, 49]. Increased attention and support for diabetes distress are recommended for females with T2DM [50].

The AR-PAID-5 scale has an acceptable criterion validity, achieving a sensitivity of 88.4%, a specificity of 95.2%, a PPV of 82.1%, and a NPV of 97.1% with cut-off value ≥8. The original PAID-5 has a sensitivity of 95% and a specificity of 89% [13].

Our study had some limitations. Lack of randomization may restrict generalization of the results, and this study included only patients with T2DM, so the results may be not generalized to patients with type 1 diabetes mellites (T1DM), thus further investigations on patients with T1DM is needed. Although the self-report method for assessing diabetes distress, depression, and anxiety can be a cost-effective and time-efficient specially at busy PHC facilities, we did not use this method of data collection as literacy rates in Egypt remain low. The PAID-5 items are obtained from the PAID. Therefore, the PAID is not an ideal external criterion for the PAID-5. A different questionnaire or a clinical interview would have been proper external criterions to classify groups with different amount of diabetes distress. The small number of patients with good glycemic control may lead to the inability of the AR-PAID-5 to confirm group-validity on achieving glycemic control targets. Also, the design of this study could not assess the responsiveness to change after interventions and needs further longitudinal study design to evaluate this. Finally recall bias during data collection may have occurred.

## Conclusions

The AR-PAID-5 scale has been demonstrated to be a psychometrically sound tool among Egyptian PHC patients with T2DM, it demonstrated good reliability and validity, and can be used as a screening tool for diabetes-related emotional distress in Egyptian PHC patients with T2DM, and may be used in other Arabic speaking patients with T2DM. This Arabic version is also relevant for use in related research to diabetes distress in Egypt or other Arabic countries. This study paves the way for future studying AR-PAID-5 scale’s utility for screening of diabetes distress in type 1 diabetes mellitus, among adults and in clinical settings other than PHC.

## Data Availability

All data generated or analyzed during this study are included in this published article.

## Abbreviations

AR-GAD-7: Arabic version of the Generalized Anxiety Disorder 7
AR-PAID: Arabic version of the Problem Areas in Diabetes
AR-PAID-5: Arabic version of the 5-item Problem Areas in Diabetes
AR-PHQ-9: Arabic version of the Patient Health Questionnaire 9
AR-WHO-5: Arabic version of the 5-item World Health Organization Well-Being Index
AUC: Area under curve
BMI: Body Mass Index
CFA: Confirmatory factor analysis
CFI: Comparative fit index
CI: Confidence interval
DDS17: 17-item Diabetes Distress Scale
DDS2: 2-item Diabetes Distress Scale
df: Degrees of freedom
GAD-7: Generalized Anxiety Disorder Scale 7
GFI: Goodness-of-fit index
HbA1c: Glycated hemoglobin
ICC: Intraclass correlation coefficient
IQR: Interquartile range
K-PAID: Korean version of the Problem Areas in Diabetes
K-PAID-5: Korean version of the 5-item Problem Areas in Diabetes
NPP: Negative predictive value
PAID: Problem Areas in Diabetes
PAID-1: 1-item Problem Areas in Diabetes
PAID-5: 5-item Problem Areas in Diabetes
PHC: Primary Healthcare
PHQ-9: Patient Health Questionnaire 9
PPV: Positive predictive value
rho: Spearman’s Rank-Order Correlation
RMSEA: Root mean squared error of approximation
ROC: Receiver operating characteristic
SD: Standard deviation
SPSS: Statistical Package for the Social Sciences
SRMR: Standardized root mean square residual
T1DM: Type 1 diabetes mellitus
T2DM: Type 2 diabetes mellitus
TLI: Tucker Lewis Index
WHO: World Health Organization
WHO-5: 5-item World Health Organization Well-Being Index
χ^2^: Chi-square
CMIN/DF: Ratio of Chi-square value to the degrees of freedom
